# Cafestol Inhibits Cyclic-Strain-Induced Interleukin-8, Intercellular Adhesion Molecule-1, and Monocyte Chemoattractant Protein-1 Production in Vascular Endothelial Cells

**DOI:** 10.1155/2018/7861518

**Published:** 2018-04-30

**Authors:** Wen-Rui Hao, Li-Chin Sung, Chun-Chao Chen, Po-Yuan Chen, Tzu-Hurng Cheng, Hung-Hsing Chao, Ju-Chi Liu, Jin-Jer Chen

**Affiliations:** ^1^Graduate Institute of Clinical Medicine, Taipei Medical University, Taipei, Taiwan; ^2^Department of Internal Medicine, School of Medicine, College of Medicine, Taipei Medical University, Taipei, Taiwan; ^3^Department of Biological Science and Technology, College of Biopharmaceutical and Food Sciences, China Medical University, Taichung, Taiwan; ^4^Department of Biochemistry, School of Medicine, College of Medicine, China Medical University, Taichung, Taiwan; ^5^Shin Kong Wu Ho-Su Memorial Hospital, Taipei, Taiwan; ^6^Department of Surgery, School of Medicine, College of Medicine, Taipei Medical University, Taipei, Taiwan; ^7^Division of Cardiology, Department of Internal Medicine, China Medical University Hospital, Taichung, Taiwan; ^8^Institute of Biomedical Sciences, Academia Sinica, Taipei, Taiwan

## Abstract

Moderate coffee consumption is inversely associated with cardiovascular disease mortality; however, mechanisms underlying this causal effect remain unclear. Cafestol, a diterpene found in coffee, has various properties, including an anti-inflammatory property. This study investigated the effect of cafestol on cyclic-strain-induced inflammatory molecule secretion in vascular endothelial cells. Cells were cultured under static or cyclic strain conditions, and the secretion of inflammatory molecules was determined using enzyme-linked immunosorbent assay. The effects of cafestol on mitogen-activated protein kinases (MAPK), heme oxygenase-1 (HO-1), and sirtuin 1 (Sirt1) signaling pathways were examined using Western blotting and specific inhibitors. Cafestol attenuated cyclic-strain-stimulated intercellular adhesion molecule-1 (ICAM-1), monocyte chemoattractant protein- (MCP-) 1, and interleukin- (IL-) 8 secretion. Cafestol inhibited the cyclic-strain-induced phosphorylation of extracellular signal-regulated kinase and p38 MAPK. By contrast, cafestol upregulated cyclic-strain-induced HO-1 and Sirt1 expression. The addition of zinc protoporphyrin IX, sirtinol, or Sirt1 silencing (transfected with Sirt1 siRNA) significantly attenuated cafestol-mediated modulatory effects on cyclic-strain-stimulated ICAM-1, MCP-1, and IL-8 secretion. This is the first study to report that cafestol inhibited cyclic-strain-induced inflammatory molecule secretion, possibly through the activation of HO-1 and Sirt1 in endothelial cells. The results provide valuable insights into molecular pathways that may contribute to the effects of cafestol.

## 1. Introduction

Cardiovascular disease (CVD) has a high mortality rate worldwide and has become a critical health concern, particularly in consideration of population aging. An epidemiological study suggested that moderate coffee consumption is inversely related to death due to CVD [1]. However, research evaluating mechanisms underlying the favorable relationship between coffee consumption and reduction in risk factors for CVD is extremely limited. Endothelial inflammation is associated with a high risk of adverse cardiovascular events [2, 3] and plays a key role in the development of CVD [4]. Several natural compounds present in coffee, such as phenolic compounds, flavonoids, and caffeic acid derivatives, have been reported to possess an anti-inflammatory property [5]. This anti-inflammatory property is likely responsible for the favorable relationship between coffee consumption and a low CVD mortality rate [6, 7]. Among the natural compounds, cafestol, a diterpene molecule found in the cherries of *Coffea arabica*, possesses various properties, including anti-inflammatory [8, 9] and antiangiogenic properties [10, 11]. However, the effect of cafestol on vascular endothelial cells remains to be clarified.

Chronic inflammation in endothelial cells produces various inflammatory mediators that exacerbate endothelial dysfunction [12]. Endothelial dysfunction caused by inflammation plays a dominant role in the pathogenesis of CVD, including atherosclerosis, hypertension, and diabetes-induced vasculopathy and vascular remodeling [13, 14]. Vascular endothelial cells are permanently exposed to mechanical stretching. Mechanical stretching, particularly cyclic strain, modulates the function of vascular endothelial cells by regulating the expression of many genes. In vascular endothelial cells, cyclic strain has been shown to increase reactive oxygen species (ROS) production, leading to the upregulation of cell adhesion molecules and cytokines [15, 16]. In addition, the cyclic straining of endothelial cells activates several proteins involved in the regulation of gene expression, including mitogen-activated protein kinases (MAPK) [17]. The MAPK family includes extracellular signal-regulated kinase (ERK), c-Jun NH_2_-terminal kinase (JNK), and p38, which are believed to be among the major regulators of proatherogenic inflammatory gene expression in endothelial cells. Adhesion molecules and inflammatory cytokines regulated by cyclic strain have been identified in endothelial cells, including intercellular adhesion molecule-1 (ICAM-1), monocyte chemoattractant protein- (MCP-) 1, and interleukin- (IL-) 8 [15, 16, 18]. Adhesion molecules and inflammatory cytokines may play a pivotal role in the pathogenesis of CVD [19]. However, the effects of cafestol on cyclic-strain-stimulated inflammatory molecule production remain unclear.

Many natural dietary compounds are believed to provide protection against oxidative stress, and a few compounds have been reported to induce genes involved in antioxidant defense through the activation of nuclear E2-related factor 2 (Nrf2) or sirtuin 1 (Sirt1) [20, 21]. The MAPK family plays an essential role in the transduction of extracellular signals to cellular responses through a cascade of phosphorylation events [22]. Cyclic strain stimulated Nrf2 expression, resulting in the subsequent expression of antioxidant enzymes, such as heme oxygenase-1 (HO-1), in stretched endothelial cells [23]. The transcription factor Nrf2 alleviates cyclic-strain-induced IL-8 expression by upregulating the expression of HO-1 [24]. We recently reported that cafestol inhibits urotensin II-induced IL-8 expression and cell proliferation via Nrf2/HO-1-dependent mechanism in endothelial cells [25]. In addition, Sirt1 is the human ortholog of the yeast silent information regulator 2 (Sir2) protein that extends the lifespan of lower organisms [26]. By interacting with several target proteins, Sirt1 performs various cellular functions including endothelial protection from vascular diseases [27]. Sirt1 exerts anti-inflammatory effects through the modulation of cytokine levels in human umbilical vein endothelial cells (HUVECs) [28]. Several natural and synthetic compounds activate Sirt1 and promote endothelial homeostasis [27, 29]. However, the effect of cafestol on MAPK, HO-1, and Sirt1 expression in cyclic-strain-activated vascular endothelial cells remains to be determined. In this study, we investigated the effects of cafestol on the modulation of cyclic-strain-stimulated inflammatory cytokine production and identified the intracellular mechanism that may be responsible for the putative effects of cafestol.

## 2. Material and Methods

### 2.1. Antibodies and Reagents

Pure cafestol (dissolved in dimethyl sulfoxide) and all other chemicals of the reagent grade were obtained from Sigma-Aldrich (St. Louis, MO, USA). All enzyme-linked immunosorbent assay (ELISA) kits were purchased from Abcam (Cambridge, UK). Antibody-directed phosphorylated ERK, phosphorylated p38, and phosphorylated JNK antibodies were obtained from Cell Signaling Biotechnology (Beverly, MA, USA). Anti-MCP-1 was purchased from Sigma-Aldrich. Anti-ERK, anti-p38, anti-JNK, anti-HO-1, anti-Sirt1, and anti-GAPDH antibodies were purchased from Santa Cruz Biotechnology (Santa Cruz, CA, USA).

### 2.2. Endothelial Cell Culture

HUVECs were obtained from PromoCell (Heidelberg, Germany), as previously described [30]. All endothelial cells used in this study were from the third to fourth passages.

### 2.3. *In Vitro* Cyclic Strain on Cultured Endothelial Cells

Endothelial cells cultured on a flexible membrane base were subjected to cyclic strain produced by a computer-controlled application of sinusoidal negative pressure, as described previously [31].

### 2.4. ELISA of Proinflammatory Molecules

For the detection of tumor necrosis factor-*α* (TNF-*α*), ICAM-1, MCP-1, IL-6, and IL-8 in the supernatant, cells were treated with and without cafestol for 12 h and then treated with cyclic strain for 24 h. After centrifugation at 1000 rpm for 10 min, the supernatant was collected to measure TNF-*α*, ICAM-1, MCP-1, IL-6, and IL-8 levels in the cell medium through ELISA. Commercially available ELISA kits (Abcam, Cambridge, UK) were used according to the manufacturer's protocol [30].

### 2.5. Intracellular ROS Analysis

Cellular ROS were analyzed using the fluorescence probe 2′,7′-dichlorodihydrofluorescein diacetate (Thermo Fisher Scientific, Waltham, MA, USA), which passively diffuses into the cell and is cleaved and oxidized to 2′,7′-dichlorofluorescein (DCF), as described previously [32].

### 2.6. RNA Extraction and Quantitative Polymerase Chain Reaction Analysis

Total RNA was extracted from vascular endothelial cells by using the TRIzol method according to the protocol recommended by the manufacturer (Thermo Fisher Scientific). The extracted RNA was used to synthesize single-stranded complementary (c)DNA by using a high-capacity cDNA reverse transcription kit (Applied Biosystems, Foster City, CA, USA), as described previously [30]. HO-1 messenger (m)RNA was quantified using TaqMan Gene Expression Master Mix (Applied Biosystems) with specific primers in an ABI 7300 Real-Time PCR System (Applied Biosystems). TaqMan gene expression assay kits containing specific primers for HO-1 and GAPDH were obtained from Applied Biosystems. Specific primers for GAPDH were used to normalize the amount of the sample added. Samples were quantified in triplicate during three separate experiments.

### 2.7. Western Blot Analysis

After each experiment, cells were washed twice with cold PBS and harvested in 150 *μ*L of lysis buffer (10 mM Tris-HCl, pH 8.0, 0.1% Triton X-100, 320 mM sucrose, 5 mM EDTA, 1 mM PMSF, 1 mg/L leupeptin, 1 mg/L aprotinin, and 2 mM dithiothreitol). Cell homogenates were centrifuged at 10,000 ×g for 20 min at 4°C. The resulting supernatant was used as a cellular protein. Samples containing 40 *μ*g cellular proteins were resolved by electrophoresis and then transferred to nitrocellulose membranes. Western blot analysis was performed as described previously [30]. The data of protein bands on Western blots were quantified using ImageJ densitometry analysis software (National Institutes of Health, Bethesda, MD, USA).

### 2.8. Sirt1 Short Interfering (si) RNA Transfection

Sirt1 siRNA and control siRNA obtained from Santa Cruz were transfected using the Lipofectamine reagent, and the experiments were performed as previously described [24].

### 2.9. Statistical Analysis

All experiments were repeated at least three times. Data are presented as the mean ± standard error of the mean. Statistical analysis was performed using Student's *t*-test or analysis of variance, where appropriate, followed by Dunnett's multiple comparison test, by using Prism Version 3.0 for Windows (GraphPad Software, San Diego, CA, USA). A *P* value of <0.05 was considered statistically significant.

## 3. Results

### 3.1. Effects of Cafestol on ICAM-1, MCP-1, and IL-8 Secretion in Cyclic-Strain-Treated HUVECs

**Figure 1:**
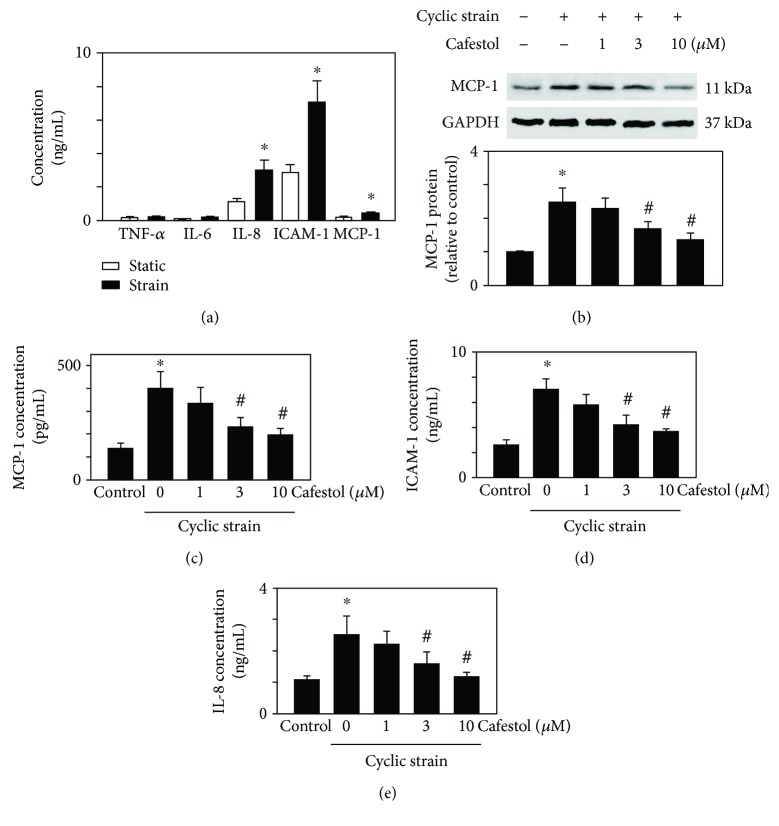
Cafestol inhibits cyclic-strain-induced inflammatory molecule secretion in HUVECs. HUVECs grown on Flexcell plates were subjected to cyclic strain for 24 h. Control cells were left under static conditions. (a) Effect of cyclic strain (−20 kPa) applied for 24 h on the production of cytokines. Values are the mean ± SEM (*n* = 3). ^∗^*P* < 0.05 versus static controls. (b) MCP-1 expression was detected using Western blot analysis with the corresponding antibody. GAPDH staining was used as a normalization control. The upper panels are representative of three independent experiments. Lower panel: the bar graph shows the fold increase in protein expression compared with static control cells. Soluble MCP-1 (c), ICAM-1 (d), and IL-8 (e) production in culture media was evaluated using ELISA; *n* = 4, ^∗^*P* < 0.05 versus the static control group. ^#^*P* < 0.05 versus the cyclic strain group.

Endothelial cells cultured on flexible membrane bases were subjected to deformation to produce an average level of strain (−20 kPa, 1 Hz). The levels of cytokines released into culture media were measured.  Figure 1(a) shows the mean levels of cytokines measured using ELISA in three separate experiments. The levels of TNF-*α* and IL-6 were not affected by cyclic strain treatment for 24 h. By contrast, the levels of IL-8, ICAM-1, and MCP-1 increased significantly after the application of cyclic strain for 24 h compared with static control cells. To evaluate the effects of cafestol on inflammatory protein expression in cyclic-strain-treated HUVECs, MCP-1 protein expression was detected using Western blot analysis. As shown in Figure 1(b), cyclic strain treatment increased MCP-1 protein expression, and cafestol (3 and 10 *μ*M) attenuated this increase in MCP-1 protein expression. Next, we evaluated the effect of cafestol on the secretion of the inflammatory proteins MCP-1, ICAM-1, and IL-8 by using ELISA. As shown in Figures 1(c)–1(e), pretreatment with cafestol (3 and 10 *μ*M) for 12 h significantly inhibited cyclic-strain-induced ICAM-1, IL-8, and MCP-1 protein secretion.

### 3.2. Antioxidative Effects of Cafestol on Cyclic-Strain-Induced ROS, ICAM-1, MCP-1, and IL-8 Production

**Figure 2:**
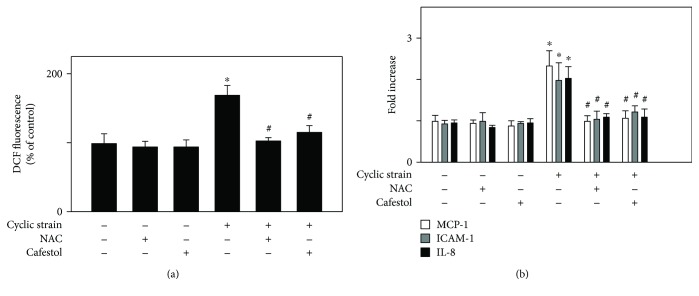
Antioxidative effect of cafestol on cyclic-strain-induced ROS production and inflammatory molecule secretion in HUVECs. Cells were pretreated with cafestol (10 *μ*M) for 12 h or NAC (10 mM) for 2 h and then exposed to cyclic strain for 24 h. (a) ROS production was assayed using DCF. (b) Protein expression levels were examined using ELISA. The bar graph shows the fold increase in protein secretion compared with control cells. Results are shown as the mean ± SEM (*n* = 5). ^∗^*P* < 0.05 versus untreated controls; ^#^*P* < 0.05 versus cells exposed to cyclic strain.

Increased ROS production in response to cyclic strain in HUVECs has been described [15, 16, 31]. Therefore, we examined ROS production in HUVECs in response to cyclic strain. Exposure to cyclic strain for 2 h led to the intracellular accumulation of ROS. Following the validation of cyclic-strain-dependent DCF fluorescence, we evaluated whether cyclic-strain-induced ROS production and inflammatory molecule protein secretion could be reduced through ROS inhibition by cafestol. As shown in Figure 2(a), the induction of ROS production by cyclic strain was prevented by pretreatment with the antioxidant *N*-acetylcysteine (NAC) and cafestol. Moreover, pretreatment with NAC and cafestol blocked the production of inflammatory molecules, including IL-8, ICAM-1, and MCP-1, in response to cyclic strain (Figure 2(b)). These results suggest that cafestol inhibits cyclic-strain-induced IL-8, ICAM-1, and MCP-1 production through ROS inhibition.

### 3.3. Cafestol Inhibits Cyclic-Strain-Activated MAPK Signaling Pathways

**Figure 3:**
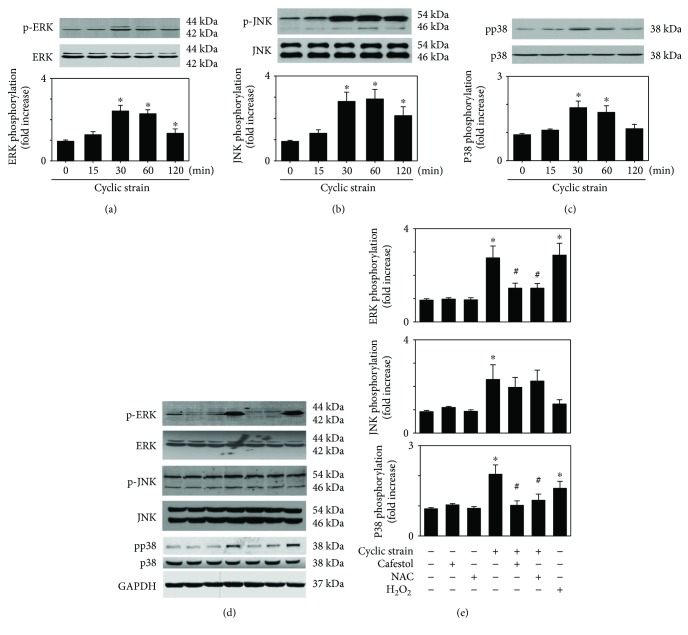
(a–c) Effects of cyclic strain on MAPK phosphorylation. Representative Western blots of phosphospecific and total ERK (a), JNK (b), and p38 (c) from cell lysates collected at the indicated times after cyclic strain treatment. Optical density measurements were obtained to determine the relative amounts of phosphorylated MAPK normalized by the respective total MAPK. The values (mean ± SEM, *n* = 4) indicate the fold change in phosphorylation relative to static controls for each individual experiment. ∗ indicates a significant difference from the static control (*P* < 0.05). (d) Effects of cafestol on cyclic-strain-induced phosphorylation of MAPK. Upper panels: Western blots of phospho-ERK, phospho-JNK, and phospho-p38 in HUVECs pretreated with cafestol (10 *μ*M) for 12 h and then treated with cyclic strain for 30 min. Lower panel: quantitative analysis of stretch-induced phosphorylation of MAKP. Results are representative of four individual experiments and expressed as the mean ± SEM (*n* = 4). ^∗^*P* < 0.05 versus untreated controls; ^#^*P* < 0.05 versus cells exposed to cyclic strain.

The cyclic straining of endothelial cells activates several proteins, including MAPK, which are believed to be among the major regulators of inflammatory gene expression [33]. HUVECs were treated with cyclic strain for different time periods, and cell lysates were immunoblotted with specific antibodies. As shown in Figures 3(a)–3(c), cyclic strain induced the phosphorylation of ERK, JNK, and p38 with a peak at 30 min in HUVECs. To investigate how cafestol affects cyclic-strain-induced MAPK phosphorylation, HUVECs were treated with 10 *μ*M cafestol for 12 h prior to cyclic strain treatment for 30 min.  Figure 3(d) shows that cafestol treatment (10 *μ*M) significantly prevented the cyclic-strain-induced phosphorylation of ERK and p38. These results indicate that the inhibition of MAPK signaling pathways may be associated with the modulatory effect of cafestol on cyclic-strain-treated HUVECs.

### 3.4. Cafestol Enhances HO-1 Expression in Cyclic-Strain-Treated Endothelial Cells

**Figure 4:**
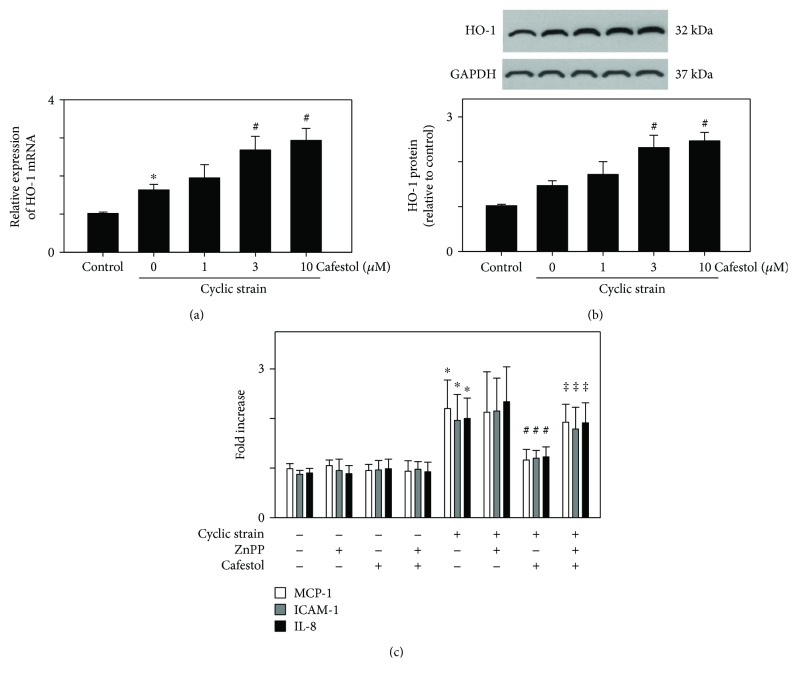
Effects of cafestol on HO-1 expression in the presence of cyclic strain treatment. HUVECs were treated with cafestol 12 h prior to cyclic strain treatment for 12 h. (a) The mRNA level of HO-1 was analyzed through qPCR and normalized to GAPDH. (b) Immunoblotting of HO-1 and GAPDH was performed, and the bands were quantitated using ImageJ. The data represent the mean ± SEM of three independent experiments. ^∗^*P* < 0.05 versus untreated control; ^#^*P* < 0.05 versus cells exposed to cyclic strain. (c) Effects of the inhibition of HO-1 on protein expression levels as examined using ELISA. HUVECs were pretreated for 30 min with Znpp, and cafestol (10 *μ*M) was then added 12 h prior to cyclic strain treatment for 24 h. Data represent the mean ± SEM of four independent experiments. ^∗^*P* < 0.05 versus untreated controls; ^#^*P* < 0.05 versus cells exposed to cyclic strain. ^‡^*P* < 0.05 versus cells exposed to cyclic strain plus cafestol treatment.

Natural products have been demonstrated to activate HO-1 to inhibit cyclic-strain-induced IL-8 expression in vascular endothelial cells [24]. We investigated how cafestol affected HO-1 expression in the presence of cyclic strain.  Figure 4(a) shows that cyclic strain treatment only slightly stimulated HO-1 mRNA expression, and pretreatment with cafestol (10 *μ*M) enhanced HO-1 upregulation.  Figure 4(b) shows that parallel to results observed in mRNA expression, the treatment of HUVECs with cyclic strain for 12 h slightly upregulated HO-1 protein expression and cafestol enhanced HO-1 protein expression. To further investigate whether decreased inflammatory molecule protein expression observed in cafestol-pretreated cells was dependent on HO-1 activity, HUVECs were treated with zinc protoporphyrin IX (ZnPP), a potent competitive inhibitor of HO enzyme activity, for 30 min, followed by cafestol for 12 h before exposure to cyclic strain for 24 h.  Figure 4(c) shows that the addition of ZnPP (1 *μ*M) attenuated the cafestol-mediated modulatory effect. These results indicate that HO-1 activity may participate in the inhibitory effect of cafestol on cyclic-strain-induced IL-8, ICAM-1, and MCP-1 production in HUVECs.

### 3.5. Effects of Sirt1 Activation by Cafestol and Sirt1 Inhibition on Cyclic-Strain-Induced Inflammatory Molecule Protein Secretion

**Figure 5:**
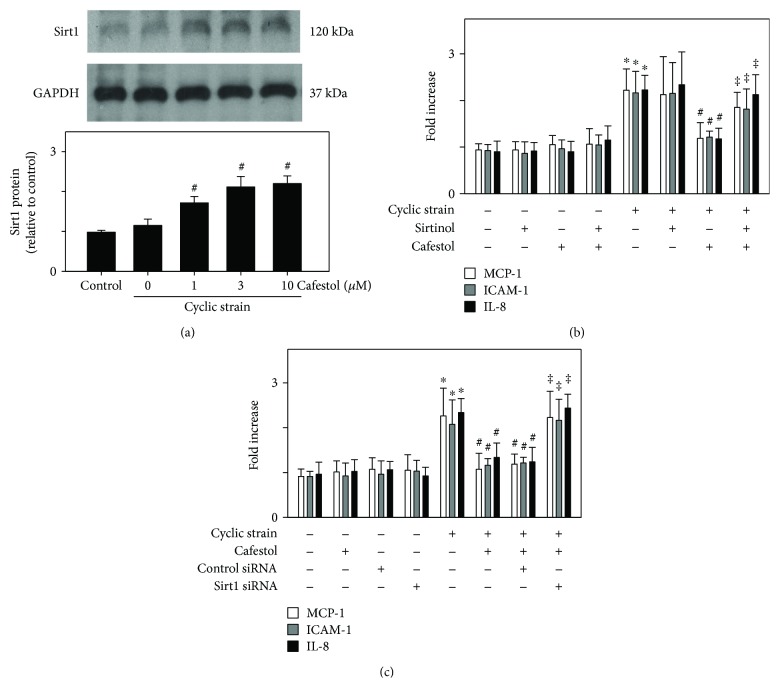
Effects of cafestol and the Sirt1 inhibitor sirtinol on cyclic-strain-induced inflammatory molecule protein expression in HUVECs. (a) Effects of cafestol on Sirt1 expression. Cells were pretreated with cafestol for 12 h or sirtinol for 2 h and then exposed to cyclic strain for 24 h. Protein expression was examined using Western blotting. The data are representative of three independent experiments. The bar graph shows the fold increase in protein expression compared with control cells. (b) Effects of the inhibition of Sirt1 by sirtinol on protein expression levels as examined using ELISA. HUVECs were pretreated with cafestol for 12 h or sirtinol for 2 h prior to cyclic strain treatment for 24 h. (c) Effects of Sirt1 siRNA on protein expression levels as examined using ELISA. Transfected cells were pretreated with 10 *μ*M cafestol for 12 h, then subjected to cyclic strain treatment for 24 h. Data represent the mean ± SEM of four independent experiments. ^∗^*P* < 0.05 versus untreated controls; ^#^*P* < 0.05 versus cells exposed to cyclic strain. ^‡^*P* < 0.05 versus cells exposed to cyclic strain plus cafestol treatment.

Accumulating evidence indicates that Sirt1 plays a crucial role in cardiovascular cell function in aging and disease [27]. A recent study showed that Sirt1 expression decreased in aged and atherosclerotic vessels *in vivo* [34]. On the basis of these results, we examined whether the modulation of inflammatory molecule production by cafestol in cyclic-strain-stimulated cells is mediated by Sirt1. Cafestol upregulated the Sirt1 protein level in cyclic-strain-treated HUVECs (Figure 5(a)). By contrast, the Sirt1 inhibitor sirtinol attenuated the induction of Sirt1 by cyclic strain but enhanced the expression of ICAM-1, IL-8, and MCP-1 proteins (Figure 5(b)). The role of Sirt1 in the inhibition of cyclic strain-induced expression of ICAM-1, IL-8, and MCP-1 by cafestol was also examined by the silencing of Sirt1. Cells transfected with Sirt1 siRNA, followed by treatment with cafestol (10 *μ*M, 12 h), abrogated the inhibitory effect of cafestol on the cyclic strain-induced expression of ICAM-1, IL-8, and MCP-1 secretion. In contrast, the control siRNA (100 nM) failed to block the inhibitory effect of cafestol. These results suggest that the effect of cafestol is linked to upregulated Sirt1 expression, and the inhibitory effect of cafestol on cyclic-strain-induced ICAM-1, IL-8, and MCP-1 protein secretion in endothelial cells at least partially depends on Sirt1.

## 4. Discussion

According to the literature review, moderate coffee consumption appears to be safe and is associated with neutral to beneficial effects on most of the studied health outcomes [35]. The major natural products in coffee that partly explain its beneficial effects are diterpenes, such as cafestol [36]. Natural products with advantages such as a potent anti-inflammatory effect and high availability have received considerable attention in recent years [37]. Inflammatory disorders usually involve many complicated mechanisms and pathways; therefore, only one drug is not adequate for treating inflammation. The use of a combination of drugs is a practical and beneficial approach. Natural products usually have multiple target interactions and have a strong therapeutic effect; therefore, combinations of natural compounds are expected to be useful in the treatment of acute and chronic inflammatory diseases in practice [38]. The role of hemodynamic forces in the pathogenesis of CVD is receiving increasing attention. Because the chemokines ICAM-1, MCP-1, and IL-8 regulate immune cell adhesion and integration with endothelial cell processes [39], they can become potential therapeutic targets. Our experiments demonstrated that among the several cytokines examined, mechanical stretching enhances the production of IL-8, MCP-1, and ICAM-1 in human endothelial cells. MCP-1, which exhibits potent monocyte chemotactic activity, is believed to be one of the crucial molecules involved in CVD [40]. The increased surface expression of ICAM-1 by cyclic-strain-activated vascular endothelial cells and local production of IL-8 may provoke leukocyte chemotaxis to the overinflated regions of the vessel and cause additional damage to the vasculature, leading to the exacerbation of vascular injury [41]. These observations may provide an explanation for an early link between the mechanical stretching of vascular walls and the prediction of CAD risk. In this study, we observed that cafestol acted as a potent inhibitor of cyclic-strain-stimulated ICAM-1, MCP-1, and IL-8 production in endothelial cells. These findings further support the anti-inflammatory effect of cafestol.

Cyclic strain induced ROS production *in vitro* [15, 16, 31]. In the current study, we found that both cafestol and NAC, a synthetic precursor of glutathione, blocked cyclic-strain-mediated ROS production, as measured by decreased DCF fluorescence. We also found that cafestol attenuated the cyclic-strain-induced phosphorylation of ERK, and p38. Cyclic strain induces ROS production and subsequently leads to the activation of MAPK signaling [42]. Therefore, it is reasonable to speculate that cafestol attenuates cyclic-strain-induced MAPK phosphorylation through the inhibition of ROS production. Apart from direct antioxidative reactivity, natural products, including cafestol, may also activate some intracellular signaling pathways, such as the Nrf2/HO-1 pathway, to prolong the cellular defense response [24, 25]. HO-1 catalyzes the rate-limiting step in heme degradation, leading to the generation of biliverdin and CO. Biliverdin and bilirubin, formed due to the action of biliverdin reductase, are potent antioxidants. In addition, CO, a major product of HO-1 activity, plays a protective role in both physiology and pathological conditions [43]. In the present study, cyclic strain alone only weakly or insignificantly induced HO-1 expression. However, cafestol pretreatment enhanced the increase in the HO-1 level, and the modulatory role of HO-1 was confirmed by the addition of the competitive inhibitor Znpp. These results provide further evidence suggesting that cafestol enhances HO-1 expression and thus scavenges excess free radicals produced by cyclic strain. Furthermore, the present data suggest that the anti-inflammatory action of cafestol may be, at least partly, due to its induction of HO-1 expression.

To date, several natural and synthetic substances, say ergothioneine, have been reported to activate Sirt1 and promote endothelial homeostasis [29, 44]. In addition, numerous studies support a cardioprotective role for sirtuin activators (e.g., resveratrol), as well as other emerging modulators of protein acetylation, including curcumin, honokiol, oroxilyn A, quercetin, epigallocatechin-3-gallate, bakuchiol, tyrosol, and berberine, and the development of sirtuin-activating compounds, such as nutraceuticals, for the management of chronic diseases has attracted considerable research interest in recent years [45]. Here we found that cafestol also enhanced Sirt1 expression in cyclic-strain-treated HUVECs. In the presence of the Sirt1 inhibitor sirtinol or Sirt1 silencing, no cafestol-mediated inhibitory effect was observed on cyclic-strain-induced ICAM-1, IL-8, and MCP-1 protein secretion. These results indicate that cafestol suppresses cyclic-strain-induced ICAM-1, IL-8, and MCP-1 protein secretion, possibly through the modulation of Sirt1 expression. Nevertheless, additional investigations are needed to fully characterize the interaction between Sirt1 and cyclic-strain-induced inflammatory protein expression.

## 5. Conclusion

Cafestol suppressed the secretion of ICAM-1, MCP-1, and IL-8 and inhibited the phosphorylation of ERK, and p38 MAPK in cyclic-strain-treated HUVECs. The mechanism of action of cafestol appears to be associated with the upregulation of HO-1 and Sirt1. On the basis of our study results, coffee consumption might be considered a preventive strategy for CVD. The results of this study might just be “the tip of the iceberg,” and additional studies are required to understand the diverse and interrelated roles of coffee consumption in disease prevention. Cafestol appears to reduce the total expression of inflammatory molecules in endothelial cells through multiple mechanisms. This study provides new insights into the anti-inflammatory properties of cafestol. The results of this study support the potential application of cafestol against inflammation-dependent disorders. The upregulation of HO-1 and Sirt1 expression and the suppression of cyclic-strain-induced ICAM-1, MCP-1, and IL-8 secretion by cafestol may be some of the possible mechanisms responsible for the protective effect of cafestol on the cardiovascular system.
