# Corrigendum to “Cafestol Inhibits Cyclic-Strain-Induced Interleukin-8, Intercellular Adhesion Molecule-1, and Monocyte Chemoattractant Protein-1 Production in Vascular Endothelial Cells”

**DOI:** 10.1155/2019/1536397

**Published:** 2019-07-04

**Authors:** Wen-Rui Hao, Li-Chin Sung, Chun-Chao Chen, Po-Yuan Chen, Tzu-Hurng Cheng, Hung-Hsing Chao, Ju-Chi Liu, Jin-Jer Chen

**Affiliations:** ^1^Graduate Institute of Clinical Medicine, College of Medicine, Taipei Medical University, Taipei, Taiwan; ^2^Division of Cardiology, Department of Internal Medicine, School of Medicine, College of Medicine, Taipei Medical University, Taipei, Taiwan; ^3^Department of Biological Science and Technology, College of Biopharmaceutical and Food Sciences, China Medical University, Taichung, Taiwan; ^4^Department of Biochemistry, School of Medicine, College of Medicine, China Medical University, Taichung, Taiwan; ^5^Shin Kong Wu Ho-Su Memorial Hospital, Taipei, Taiwan; ^6^Department of Surgery, School of Medicine, College of Medicine, Taipei Medical University, Taipei, Taiwan; ^7^Division of Cardiology, Department of Internal Medicine, China Medical University Hospital, Taichung, Taiwan; ^8^Institute of Biomedical Sciences, Academia Sinica, Taipei, Taiwan

In the article titled “Cafestol Inhibits Cyclic-Strain-Induced Interleukin-8, Intercellular Adhesion Molecule-1, and Monocyte Chemoattractant Protein-1 Production in Vascular Endothelial Cells” [1], there were errors in the first and second affiliations. The corrected affiliations' list is shown above.
